# Impact of different assumptions on estimates of childhood diseases obtained from health care data: A *retrospective cohort study*

**DOI:** 10.1002/pds.4413

**Published:** 2018-04-24

**Authors:** Osemeke U. Osokogu, Alexandra Pacurariu, Mees Mosseveld, Peter Rijnbeek, Daniel Weibel, Katia Verhamme, Miriam C.J.M. Sturkenboom

**Affiliations:** ^1^ Department of Medical Informatics Erasmus University Medical Center Rotterdam Netherlands; ^2^ University Medical Center Utrecht Julius Center The Netherlands

**Keywords:** children, incidence, methodology, pharmacoepidemiology, prevalence

## Abstract

**Purpose:**

Accurate estimates of disease incidence in children are required to support pediatric drug development. Analysis of electronic health care records (EHR) may yield such estimates but pediatric‐specific methods are lacking. We aimed to understand the impact of assumptions regarding duration of disease episode and length of run‐in period on incidence estimates from EHRs.

**Methods:**

Children aged 0 to 17 years (5–17 years for asthma) registered in the Integrated Primary Care Information database between 2002 and 2014 were studied. We tested the impact of the following: maximum duration of disease episode (0, 14, 30, 60, and 90 days) on recurrent diseases (acute otitis media [common] and acute pyelonephritis [rare]); and database run‐in period on chronic diseases—asthma (common) and type 1 diabetes (DM) (rare). We calculated incidence rate ratios with 95% confidence intervals and stratified using 1‐year age categories.

**Results:**

Altogether, 503 495 children were registered. The incidence of acute otitis media was highest in <2‐year‐old children; using 30 days disease duration as reference, the rate increased with 8% if the duration was 14 days and decreased with 8% when extended to 60 days. Disease duration did not impact acute pyelonephritis (rare). No run‐in (to exclude prevalent cases) versus 24‐month run‐in period overestimated the incidence rate for asthma and DM by a factor of 2.

**Conclusions:**

Analysis of EHR allows for estimation of disease incidence in children, but assumptions regarding episode length and run‐in period impact the incidence estimates. Such assumptions may be routinely explored.

## 1 INTRODUCTION

Globally, legislations have been introduced to stimulate development of drugs for children specifically.^1^, ^2^, ^3^ Regulatory authorities now require pharmaceutical companies to include pediatric investigation plans (PIPs) when submitting proposals for drug development in adults. PIPs may be waived if the target indication affects only adults. For PIPs that are considered for approval, the potential therapeutic benefits for children should be explained in the document. Such explanation may include data regarding the background occurrence of the indication in the pediatric population.^4^

Population‐based electronic health care records (EHR) provide an excellent data source for estimation of disease occurrence;^5^ however, there are specific methodological challenges that should be considered based on the fact that these data are not collected for research but for every day care.

First, since EHR was introduced only in the last few decades, software systems may change, and patients may move between physicians/health care plans. Therefore, the data often capture only a specific (limited) part of an individual's life‐time. In order to distinguish incident from prevalent disease, researchers usually apply a look‐back (run‐in) period which is often arbitrarily chosen and the impact of the choice is not investigated or reported.^6^ In addition, patients (or their parents) visit a physician usually at the start of a disease but not anymore once the disease has resolved, which hampers calculation of the duration of transient diseases.

Secondly, the characteristics of childhood diseases present additional challenges; children suffer from mostly common transient infection‐related disease such as acute otitis media (AOM). On the other end of the spectrum of recurrent diseases, there may be rare diseases like acute pyelonephritis (APN).^7^, ^8^ Diseases that affect children may also be chronic and differ in frequency: asthma is common and chronic unlike type 1 diabetes (DM) which is also chronic but less common.^9^, ^10^ It would be important to understand the impact of different assumptions on estimates of disease occurrence.

As part of the Global Research in Pediatrics—Network of Excellence (http://www.grip-network.org/), we aimed to understand how different assumptions regarding duration of a disease episode (for transient and recurrent diseases) and run‐in period (for chronic diseases) may impact incidence and prevalence estimates. As examples, we investigated AOM and APN (both transient and recurrent), and asthma and type 1 diabetes (both chronic conditions).

**KEY POINTS**

- Limitations arising from lack of standardized methodologies to calculate incidence of disease in children may lead to heterogeneity in reported incidence data that are required for the pediatric investigation plans.
- As part of the Global Research in Pediatrics, we demonstrate the impact of applying different assumptions regarding duration of disease episode and run‐in period and provide recommendations for dealing with these.

## 2 METHODS

### 2.1 Setting

This retrospective cohort study was performed using the Integrated Primary Care Information (IPCI) database as an example for other electronic health care databases. IPCI is a longitudinal, observational, primary care database with records of approximately 1 500 000 patients from approximately 450 general practitioners (GPs) in the Netherlands. The records comprise information on patient demographics, symptoms and diagnoses, referrals, laboratory tests and results, drug prescriptions, hospitalizations, and discharge letters. Details of the data source have been published elsewhere.^11^ Diagnoses are coded according to the International Classification for Primary Care (ICPC).^12^ Drug names are coded following the World Health Organization‐Anatomic Therapeutic Chemical (WHO‐ATC) classification system. The database has been proven to be valid for conducting pharmacoepidemiological studies.^13^

### 2.2 Study population

All children aged 0 to 17 years that were registered for at least 1 day between January 1, 2002 and December 31, 2014 could be included in the study. For the investigation of asthma, the minimum age for inclusion was 5 years because the diagnosis of asthma in children under 5 years old is prone to misclassification due to the high incidence of viral infections associated with wheezing.^14^, ^15^ Patients entered the study population at the latest of the following dates: start of the study period, date of birth or date of registration in IPCI, age of 5 years (asthma only). For both asthma and type 1 diabetes, patients needed to have up to 24 months' run‐in to be included in the study population.

Exit from the study population occurred at the earliest of the following events: leaving the GP practice, death, subject turned 18 years old, or end of the study period.

### 2.3 Outcome definition and identification

Four outcomes of interest were studied based on different durations (transient or chronic) and frequencies (common or rare). The outcomes were identified based on diagnosis and prescription codes. See appendix 1.

Acute otitis media (AOM) is a transient disease, the systemic and local features of AOM usually resolve within 24 to 72 hours.^16^, ^17^ One patient can experience more than 1 episode of AOM.^18^ Children with AOM were identified through a search on the ICPC AOM disease code H74.

Acute pyelonephritis (APN) is also a transient disease. In the Netherlands, ICPC disease code U70 implies that APN was diagnosed by urine testing.^19^ Also, this code distinguishes APN from cystitis which is assigned the ICPC disease code U71 thereby preventing misclassification of both forms of urinary tract infection (UTI). APN may be recurrent.^20^

Asthma is a chronic and rather common condition in children.^10^ Cases were identified by combining the ICPC disease code (R96) with at least 2 prescriptions for asthma medication (ATC code R03) in the year following the initial diagnosis.^10^, ^21^

Type 1 diabetes (DM) is a chronic and rare disease in children. Cases were identified by combining ICPC disease code (T90) and at least 1 prescription for insulin (A10A) in the year following the initial diagnosis.^22^

### 2.4 Statistical analyses

Overall and age‐specific incidence rates (IR) were calculated by dividing the events/outcomes by the total number of person‐years (PY) accumulated in the study population. The IR was expressed per 100 000 PY. Age stratification was done in 1‐year categories. For the transient outcomes (AOM and APN), recurrent episodes were considered as new events based on a duration of episode of 0, 14, 30, 60, and 90 days. Person‐time was not censored at diagnosis. For the chronic outcomes, person‐time was censored at the date of first diagnosis. The run‐in period was reduced from 24 to 12 and 6 months to assess the impact on the incidence rate. Further, the impact of not applying a run‐in period was tested. The 95% confidence intervals (CI) around the incidence rates were estimated based on the negative binomial distribution.^23^ For the transient outcomes (AOM and APN), age‐specific incidence rate ratios (IRR) were calculated by comparing the IRs based on clinically plausible episode durations—14 days vs 30 days. For the chronic outcomes (asthma and DM), age‐specific IRRs were calculated by dividing the IR resulting from not applying run‐in vs 24 months' run‐in.

**Figure 1:**
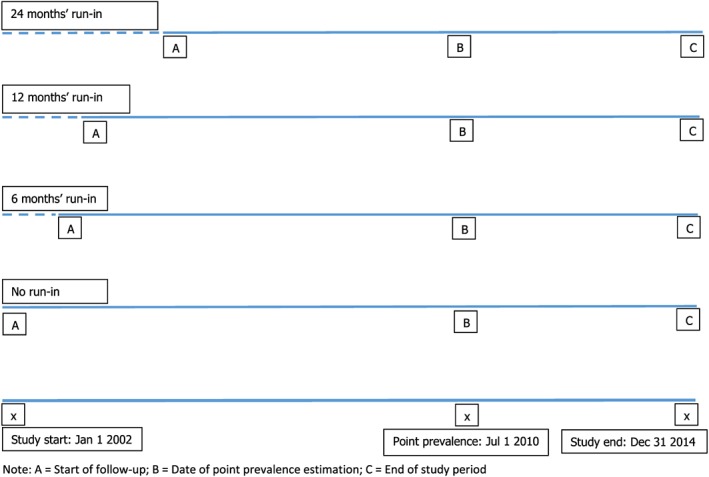
Study schematic showing the run‐in periods, start of follow‐up and timing of outcome definitions [Colour figure can be viewed at http://wileyonlinelibrary.com]

As presented in Figure 1, point prevalence was calculated on July 1, 2010 by dividing the number of children with the outcome on that date by the total number of children in the study population on that date. 95% CIs were calculated based on the Wilson score interval.^24^

To calculate the age‐specific prevalence ratio (PR) for the transient outcomes (AOM and APN), we assumed an event to be new if it occurred ≥30 days after the preceding event, and we divided the resulting point prevalence by the estimate that was based on 14 days' episode duration. Regarding the chronic outcomes (asthma and DM), age‐specific PRs were calculated by dividing the point prevalence resulting from 24 months' run‐in vs no run‐in. For the calculation of the point prevalence based on the 24 months' run‐in, this meant that only those children with a database history of at least 24 months could be included in the denominator. Of these children, only those that met the case definition (as described earlier) prior to July 1, 2010 were included in the numerator. No run‐in meant that no database history was required for inclusion. The 95% CIs around the IRRs and PRRs were calculated following the negative binomial distribution.^25^

Analyses were conducted using a custom‐built Java application called Jerboa Reloaded (Erasmus University Medical Center, Rotterdam), and IBM SPSS Statistics for windows version 21.0, Armonk, NY: IBM Corp.

## 3 RESULTS

**Figure 2:**
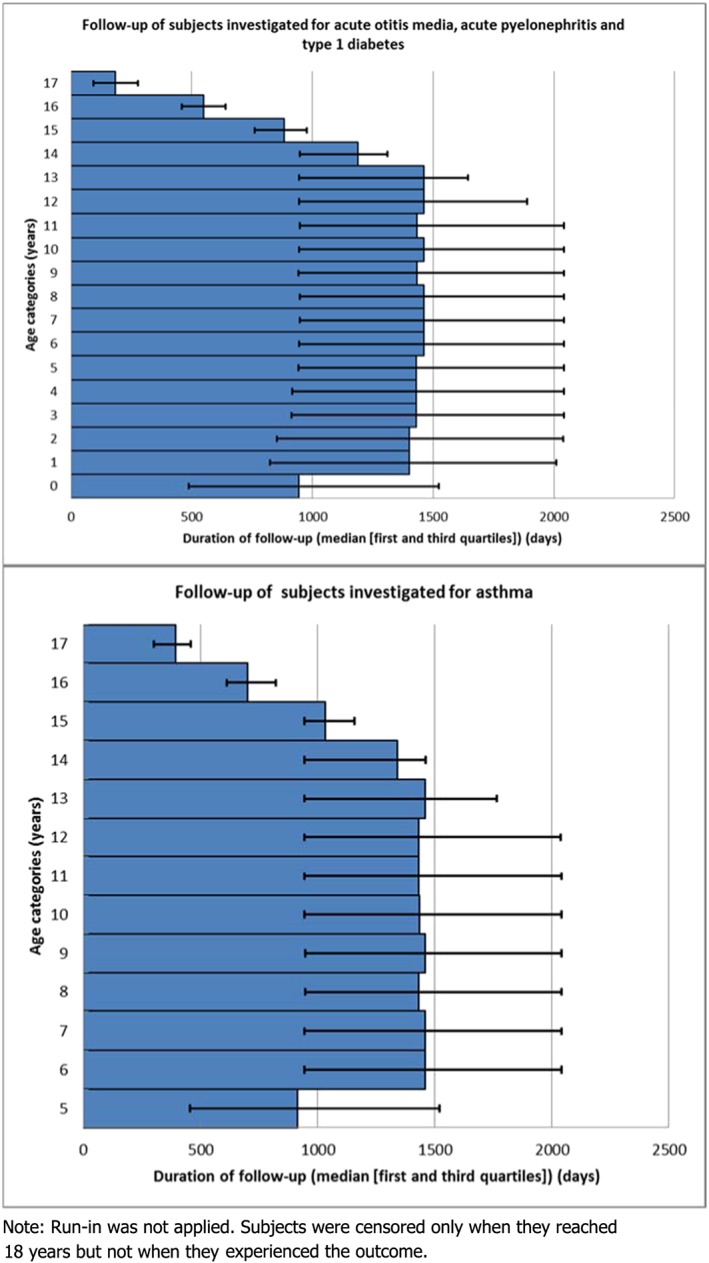
Median follow‐up time according to the age categories of the studied populations [Colour figure can be viewed at http://wileyonlinelibrary.com]

To investigate AOM and APN, the study population comprised 503 495 children. For asthma (studied in children 5 years or older) and DM, we studied 304 856 and 405 600 children, respectively; the total PYs of follow‐up were 710 980 PY and 1 042 067 PY. Figure 2 shows the distribution of age (at start of follow‐up [no run in]) and duration of follow‐up of the population base, without censoring.

#### Recurrent diseases and impact of episode duration

Based on the assumptions that a new event can only re‐occur 0, ≥14, ≥30, ≥60, or ≥90 days after the preceding event, overall IRs for AOM decreased from 8.2, 7.1, 6.6, 6.2 to 5.9 per 100 PY, respectively (Table 1).

**Table 1:** Total number of studied children, total person‐years (PY) of follow‐up, total number of incident events (transient and recurrent outcomes) or cases (chronic outcomes) and overall incidence rates according to the investigated outcomes

Outcome	Assumption a	Total Number of Subjects b	Total Person‐Years (PY)	Total Number of Events/Cases	Incidence Rate (per 100 000 PY)
Acute otitis media	0 days	503 495	1 781 625	146 391	8216.7
	≥14 days	503 495	1 761 172	124 749	7083.3
	≥30 days	503 495	1 752 235	115 107	6564.0
	≥60 days	503 495	1 746 245	107 860	6176.7
	≥90 days	503 495	1 742 821	103 089	5915.1
Acute pyelonephritis	0 days	503 495	1 734 774	540	31.1
	≥14 days	503 495	1 734 750	513	29.6
	≥30 days	503 495	1 734 740	502	28.9
	≥60 days	503 495	1 734 724	484	27.9
	≥90 days	503 495	1 734 713	468	27.0
Asthma^b^	No run‐in	304 856	710 980	4238	596.1
	6‐month run‐in	304 856	710 980	3385	476.1
	12‐month run‐in	304 856	710 980	2786	391.9
	24‐month run‐in	304 856	710 980	1881	264.6
Type 1 diabetes	No run‐in	405 600	1 042 067	256	24.6
	6‐month run‐in	405 600	1 042 067	212	20.3
	12‐month run‐in	405 600	1 042 067	172	16.5
	24‐month run‐in	405 600	1 042 067	115	11.0

a For the transient outcomes, this refers to the time between new episodes; for the chronic outcomes, it refers to the length of the run‐in period.

b For asthma and type 1 diabetes, subjects that had a minimum 24‐month run‐in were studied to know the impact of decreasing the run‐in period on the incidence rate

**Figure 3:**
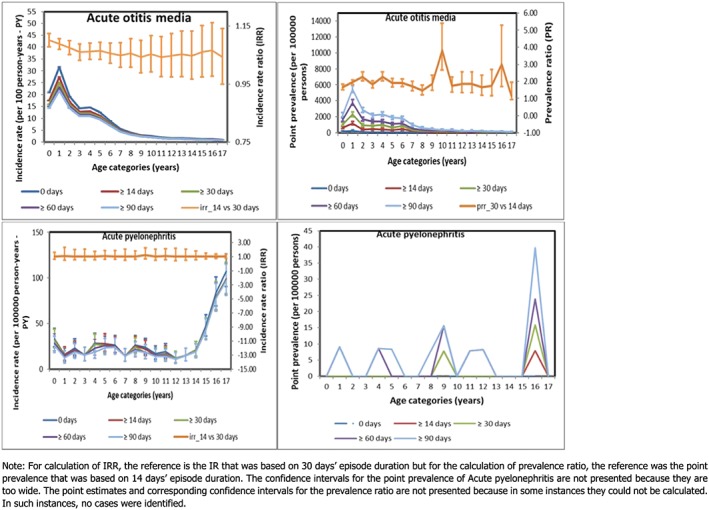
Incidence rate, incidence rate ratio, point prevalence and prevalence ratio for the transient outcomes [Colour figure can be viewed at http://wileyonlinelibrary.com]

The estimates resulting from the shortest assumed duration were the highest, decreasing with increasing length of an episode in all age categories (Figure 3).

Incidence was highest in the youngest children although this did not affect the impact of episode duration on the IRR: comparing 14 vs 30 days: IRR = 1.10 (95% CI: 1.08; 1.12) for subjects aged <1 year and 1.09 (95% CI: 1.07; 1.11) for subjects aged 1–2 years; overall IRR = 1.08 (95% CI: 1.07; 1.09). The IRs for APN were much lower than for AOM. Assuming 0, ≥14, ≥30, ≥60, or ≥90 days between new events, the overall IRs reduced relatively little from 31.1, 29.6, 28.9, 27.9 to 27.0 per 100 000 PY, respectively. The age‐specific IRRs to test assumptions on duration of episodes comparing 14 vs 30 days were all around 1, showing no age specific effect modification of the impact of episode duration.

The impact of episode duration length had quite an opposite effect on the point prevalence: with increasing duration of episodes, the point prevalence increased. The overall PRR that compared an episode duration of 30 days to 14 days' duration for AOM was 1.92 (95% CI: 1.73; 2.12), which was higher than for APN: 1.50 (95% CI: 0.25; 8.98). In APN, the impact was less pronounced due to a very low prevalence overall.

#### Chronic diseases and impact of run‐in/naïve period

**Figure 4:**
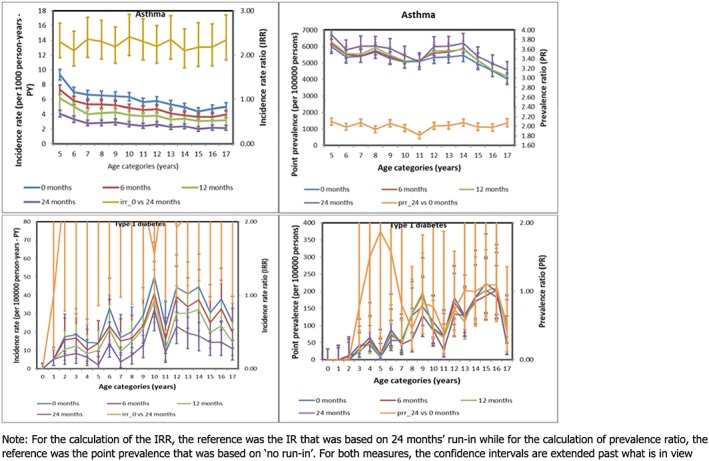
Incidence rate, incidence rate ratio, point prevalence, and prevalence ratio for the chronic outcomes [Colour figure can be viewed at http://wileyonlinelibrary.com]

By applying a 0, 6, 12, or 24 months' naïve period, overall IRs for asthma (age 5–17 years) lowered from 5.96, 4.76, 3.92 to 2.65 per 1000 PY, respectively. The impact of a 0 vs 24 months' run‐in was a more than 2‐fold increase in IR across all ages (Figure 4), IRR overall = 2.25 (95%CI: 2.13; 2.38).

By applying a 0, 6, 12, or 24 months' naïve period, overall IRs for DM were 2.46, 2.03, 1.65, and 1.10 per 10 000 PY, respectively. Again, the highest IRs were observed when no run‐in was applied and the IRs were lowest with 24‐months' run‐in. The impact of applying 0 vs 24 months' run‐in leads to a 2‐fold increase in incidence rate in most age categories, IRR overall = 2.22 (95%CI: 1.79; 2.77). The impact of an increase in the run‐in period, during which prevalent cases would be identified and excluded, was a lowering of the rate of asthma as well as DM.

The impact of applying a 24 months' run‐in vs no run‐in on the age specific point prevalence was negligible for both asthma (overall PRR: 1.10 [95% CI: 1.06; 1.14]) as well as DM (overall PRR: 0.82 [95% CI: 0.64; 1.04]).

**Figure 5:**
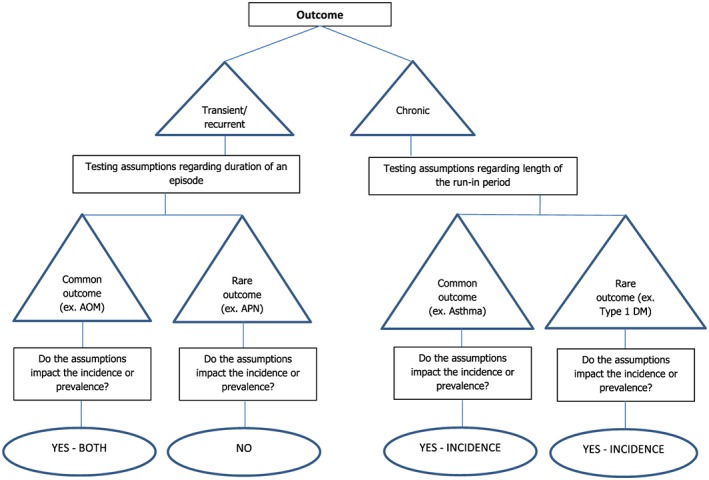
Summary of the impact of assumptions on the investigated outcomes [Colour figure can be viewed at http://wileyonlinelibrary.com]

The results are summarized in Figure 5.

## 4 DISCUSSION

This study showed that assumptions regarding duration of a disease episode (for transient and recurrent diseases) and run‐in period (for chronic diseases) may impact incidence and prevalence estimates of childhood diseases obtained from population‐based dynamic EHR databases. While this study was focused on children, some of the investigated issues may also be relevant for adults. The lack of complete follow‐up from birth till 17 years of age, and the fact that only the visit for a disease and not the ending of a disease is recorded in the electronic medical record has an impact on estimation of disease occurrence. Usually, epidemiologists apply assumptions to deal with these limitations, such as “assuming a standard disease episode duration” and use of a run‐in period prior to start of follow‐up that can be used to exclude prevalent disease. In this paper, we wanted to investigate the impact of these assumptions on the different occurrence measures in children, and we witnessed relatively great impact. General rules can be obtained from this exercise: for common recurrent diseases, the impact of the choice of episode duration is relatively high, assuming longer disease episodes leads to lower incidence. The impact of a change in episode duration on the incidence is negligible in a rare recurrent disease. This is understandable because the probability of having another event of a rare disease is low, and this will not likely occur close together. The impact of an increasing episode duration on the prevalence of a common recurrent disease is opposite, with increasing duration the point prevalence increases. The impact is negligible on the point prevalence for a rare recurrent disease. We recommend that studies aiming to estimate incidence and prevalence of common recurrent diseases better explore the impact of the episode length because the true length cannot be observed in medical record databases. Patients do not return to tell the GP that disease is cured. For rare diseases, the impact of different episode durations may be ignored both for incidence and prevalence estimations. For chronic diseases, varying the run‐in impacts the incidence with negligible impact on the prevalence. We recommend that studies investigating chronic diseases apply the longest possible run‐in to avoid misclassifying prevalent cases as incident. We admit that this may lead to a reduced sample size and depending on the database can potentially limit the generalizability of the results.

Regarding AOM, there was no difference in incidence when we compared 2 clinically plausible episode durations: 14 versus 30 days; 30 days has been applied in a previous study.^7^ Based on the natural history of AOM, the actual duration of an episode is not clear.^26^ Therefore, we also compared the incidence rates (results not presented) we derived from the shortest (0 days) versus longest (90 days) assumptions; the estimates were significantly different. When we performed the same comparisons for APN, the result was not significant, further confirming that the episode duration is important for estimating the occurrence of only the common outcomes. We observed that assuming episode duration of 30 vs 14 days significantly impacted the prevalence of AOM. The peak in PR that was observed among children aged 9 to 10 years is probably due to the comparatively lower number of events in that age group. To derive the most clinically meaningful estimate of incidence and/or prevalence of AOM and to a less extent APN, perhaps the most plausible assumption for the duration of an episode remains 30 days. It is highly unlikely that an episode will last for as short as a few hours to 1 day or as long as 90 days. We recommend further research to know the most appropriate assumptions to apply when estimating the occurrence of AOM and other common and recurrent childhood diseases. Prevalence might be a good measure.

Still regarding AOM, the IRR seemed consistent across all ages, but the absolute difference in IR estimates between the assumed disease durations would be much smaller for older compared with younger children. In older children, there is minimal or no difference in incidence estimates regardless of the assumed duration of an episode.

Regarding both asthma and DM, increasing the run‐in period considerably decreased the incidence. This finding is important given that people can be observed for only a part of their lifetime; despite conducting the current study over a 12‐year calendar period, the median duration of follow‐up for the studied population was 1500 days, showing the incomplete follow‐up (Figure 2). Therefore, we recommend that the longest feasible run‐in period be applied when estimating the incidence of chronic diseases. It is expected that the point prevalence of chronic conditions should increase with a longer run‐in period. However, the results for DM did not reflect this. Rather, overall PR for DM, comparing 24 months' vs no run‐in was 0.82 (95% CI: 0.64; 1.04). This finding may have resulted from the fact that type 1 DM is rare in children, and therefore the estimates we derived for this condition are unstable. Further research is required.

Although this is a methodological study that was primarily aimed at testing assumptions rather than the incidence or prevalence estimates themselves, it is important to note that contrary to what is expected, the prevalence of asthma did not increase with increasing age. This finding may have resulted from the following possible scenarios: first, older children may have seen the physician less frequently; secondly, the case definition we applied (based on previous studies) may have inadvertently excluded newly diagnosed cases in older children because we required at least 2 prescriptions for asthma medications in the year following the initial diagnosis; thirdly, the fact that the study population was dynamic may have contributed to this finding. Perhaps, conducting this study within a fixed cohort or using all the available look‐back information would have led to different results. Further research is required.

This study has strengths and limitations. As strengths, we tested assumptions that are plausible from both an epidemiological and clinical point of view, and we demonstrated the ability to estimate the impact of different assumptions. The limitations include the following: first, we investigated only 4 different conditions and tried to draw general conclusions. However, we believe the conclusions hold but cannot provide thresholds when a disease or condition is considered rare or common. This will be a continuum; with the specific demonstrators, we tried to show that assumptions should be considered and the impact may be considerable. Secondly, the case definitions for asthma and DM were aimed at increasing the likelihood of identifying confirmed cases rather than cases with a provisional diagnosis. This may have certain implications. Frequency measures may be underestimated for patients that are lost to follow‐up in the year following the initial diagnosis or those that were first diagnosed at 17 years but censored when they turn 18 years. Thirdly, there are conditions like depression with insidious onset. For such conditions, more complex definitions are required for identification of more relevant assumptions regarding disease duration and run‐in. Fourthly, we applied the assumptions to only 1 database, a highly dynamic GPs' database. The impact of the episode length is generalizable across all databases, but the impact of the run‐in period may be less pronounced in more stable regional or national databases where persons are registered from birth. Lastly, the IPCI database is a GP database and therefore may impact the identification of diseases or conditions that are usually diagnosed by the specialist. However, the database contains information on referrals and hospitalizations, and therefore it is expected that the aforementioned impact will be negligible.

## 5 CONCLUSIONS

Population based EHR provide a rich and readily available source of data for estimation of disease occurrence in children which can be used in PIPs. Trial planning as well as potential market implications usually requires estimates of occurrence in children. Assumptions on the episode length and run in period may impact a lot on the absolute measures of occurrence and should be explored, especially in more common childhood diseases.

## FUNDING

The Global Research in Pediatrics—Network of Excellence is funded under the European Union's Seventh Framework Programme (FP7/2007–2013) for research, technological development, and demonstration under grant agreement number 261060. Funding for this study was also received from the “Priority Medicines Kinderen project ZONMW: EVIPED: Novel methods to assess and compare drug effects in pediatrics” (Grant agreement number 113201007). The funders had no role whatsoever in designing and conducting the study, the collection and management of data, and preparation, review, or approval of the manuscript.

## CONFLICT OF INTEREST

M.S. is heading a research group that occasionally conducts post‐authorization safety studies for pharmaceutical companies; none is related to this topic. K.V. has received unconditional grants from Pfizer/Boehringer Ingelheim, Yamanouchi, Novartis, and GlaxoSmithKline; none are related to the contents of this paper. P.R. has participated in studies that were funded by unconditional grants from Novartis, GlaxoSmithKline, Johnson and Johnson; none is related to the contents of this paper. A.P. is an employee of Dutch Medicines Evaluation Board. The views expressed in this article are the personal views of the author(s) and may not be understood or quoted as being made on behalf of or reflecting the position of the Dutch Medicines Agency.

O.O. and D.M. have no conflicts of interest to declare.

## ETHICS STATEMENT

The study and the access to the database were approved by the IPCI governing board (number 05/2015).

## Supporting information


**Appendix 1**: Characteristics and event (transient and recurrent outcomes) or case (chronic outcomes) definitions for the investigated outcomes
