# Supplementary material for: Impact of different assumptions on estimates of childhood diseases obtained from health care data: A retrospective cohort study
Source: Pharmacoepidemiol Drug Saf. 2018 Apr 24;27(6):612–20. doi: 10.1002/pds.4413 (PMC6001570; doi:10.1002/pds.4413)
Supplement: Supplementary file 1 — Appendix 1: Characteristics and event (transient and recurrent outcomes) or case (chronic outcomes) definitions for the investigated outcomes [file PDS-27-612-s001.docx]

**Appendix 1: Characteristics and event (transient and recurrent outcomes) or case (chronic outcomes) definitions for the investigated outcomes**

| Outcome | Duration | Frequency | Prevalence/Incidence in general population | Clinical definition | Case definition | Alternative assumptions |
| --- | --- | --- | --- | --- | --- | --- |
| Acute otitis media: | Transient | Common | 107 episodes/1000 PY[^1^](#_ENREF_27) | ‘Acute otitis media is understood to be an inflammation of the middle ear with a maximum duration of three weeks. Acute otitis media is generally associated with earache, symptoms of general illness, fever and sometimes purulent discharge (otorrhoea), and is characterised by a bulging tympanic membrane with change in colour (red or opaque)’ [^2^](#_ENREF_28) the systemic and local features of AOM usually resolve within 24-72 hours [^3^](#_ENREF_28)^,4^. | ICPC code H71[^5^](#_ENREF_28) | Varying the time between new events ^a^  0 days  ≥14 days  ≥30 days  ≥60 days and  ≥90 days |
| Acute pyelonephritis | Transient | Rare | 15.7/100000 persons[^6^](#_ENREF_28) | Symptoms of Urinary Tract Infection (UTI) in children may include fever, vomiting, screaming, anorexia, and irritability. Acute pyelonephritis is a component of UTI [^7^](#_ENREF_28) | ICPC code U70^b^ AND prescription of any antibiotic (J01) on the same date of diagnosis^c^ ^7^ | Varying the time between new events ^b^  0 days  ≥14 days  ≥30 days  ≥60 days  ≥90 days |
| Asthma | Chronic | Common | 6.7/1000 PY^8^ | Asthma is a syndrome with a highly variable clinical spectrum, characterised by airway inflammation ^9^ | ICPC code R96 AND at least two prescriptions for asthma medications (R03) in the first year following the initial diagnosis [^8^](#_ENREF_10). | Varying the run-in period^d^  No run-in (prevalent cases included)  6 months  12 months  24 months |
| Type 1 diabetes | Chronic | Rare | 23.2/100000 children [^10^](#_ENREF_9) | ‘Type 1 diabetes is an autoimmune –mediated disease associated with several complications and decrease in quality of life’ [^11^](#_ENREF_31) | ICPC code T90 AND at least one prescription of insulin (A10A) in the first year following the initial diagnosis [^12^](#_ENREF_22). | Varying the run-in period^d^  No run-in (prevalent cases included)  6 months  12 months  24 months |

^a^ For ‘0 days’, this implies that every record is considered as a new episode and for the other assumptions, all records within the stated time window (<14 , <30 , <60, and <90 days) are considered as the same episode.

^b^ According to the Dutch classification, ICPC code U70 implies that APN was diagnosed by urine testing [^7^](#_ENREF_19)

^c^ In the Netherlands, physicians are encouraged to administer antibiotics for every case of AP because of the risk of renal scarring.

^d^ This applies only to prevalence calculation; run in period will not be applied to patients with age< run-in period

**References**

1. Plasschaert AIO, Rovers MM, Schilder AGM, Verheij TJM, Hak E. Trends in doctor consultations, antibiotic prescription, and specialist referrals for otitis media in children: 1995–2003. *Pediatrics*. 2006;117(6):1879‐1886.

2. NHG Clinical Practice Guidelines. NHG Clinical Practice Guidelines. M09 Acute Otitis Media (AOM). M29 Febrile Illnes in Childeren 2011; <http://assortiment.bsl.nl/files/e27c6c2f‐8fa3‐4f91‐9c60‐>39597b8ecf7d/voorbeeldhoofdstuk.pdf. Accessed 27th October, 2015.

3. Glasziou PP, Del Mar CB, Sanders SL, Hayem M. Antibiotics for acute otitis media in children. *Cochrane Database Syst Rev (Online)*. 2004;(1):CD000219.

4. Rovers MM, Glasziou P, Appelman CL, et al. Antibiotics for acute otitis media: a meta‐analysis with individual patient data. *Lancet*. 2006;368(9545):1429‐1435.

5. Uijen JH, Bindels PJ, Schellevis FG, van der Wouden JC. ENT problems in Dutch children: trends in incidence rates, antibiotic prescribing and referrals 2002‐2008. *Scand J Prim Health Care*. 2011;29(2):75‐79.

6. Harmsen M, Wensing M, Braspenning JCC, Wolters RJ, Van Der Wouden JC, Grol RPTM. Management of children's urinary tract infections in Dutch family practice: a cohort study. BMC Fam Pract. 2007;8. ((Harmsen M., m.harmsen@kwazo.umcn.nl; Wensing M., m. wensing@kwazo.umcn.nl; Braspenning J.C.C., [j.braspenning@kwazo](mailto:j.braspenning@kwazo). umcn.nl; Wolters R.J., r.wolters@kwazo.umcn.nl; Grol R.P.T.M., r. grol@kwazo.umcn.nl) Centre for Quality of Care Research (WOK), Radboud University Nijmegen Medical Centre, 6500 HB Nijmegen, Netherlands)

7. Harmsen M, Wensing M, Braspenning J, Wolters R, van der Wouden J, Grol R. Management of children's urinary tract infections in Dutch family practice: a cohort study. *BMC Fam Pract* 2007/03/13. 2007;8(1):1‐7.

8. Engelkes M, Janssens HM, de Ridder MAJ, de Jongste JC, Sturkenboom MCJM, Verhamme KMC. Time trends in the incidence, prevalence and age at diagnosis of asthma in children. *Pediatr Allergy Immunol*. 2015;26(4):367‐374.

9. Brand PLP, Baraldi E, Bisgaard H, et al. Definition, assessment and treatment of wheezing disorders in preschool children: an evidence based approach. *Eur Respir J.* 2008;32(4):1096‐1110.

10. Spaans EAJM, van Dijk PR, Groenier KH, et al. Seasonality of diagnosis of type 1 diabetes mellitus in the Netherlands (Young Dudes‐2). *J Pediatr Endocrinol Metab.* 2016;29(6):657‐661.

11. Spaans EAJM, Gusdorf LMA, Groenier KH, et al. The incidence of type 1 diabetes is still increasing in the Netherlands, but has stabilised in children under five (Young DUDEs‐1). *Acta Paediatr.* 2015;104(6):626‐629.

12. Fazeli Farsani S, Souverein PC, Vorst MM, et al. Increasing trends in the incidence and prevalence rates of type 1 diabetes among children and adolescents in the Netherlands. *Pediatric diabetes.* 2016 Feb 1;17(1):44-5
